# Repurposing GLP-1 receptor agonists for alcohol use disorder: a systematic review and meta-analysis

**DOI:** 10.1186/s13098-025-02006-x

**Published:** 2026-01-08

**Authors:** Amir Nasrollahizadeh, Ghazaleh Kheiri, Sepide Javankiani, Sadra Kheiri, Seyedeh Fatemeh Hamzavi, Mehdi Karimi, Ehsan Amini-Salehi, Mohammad Amin Karimi

**Affiliations:** 1 Cardiovascular Diseases Research Institute, Tehran Heart Center, Tehran University of Medical Sciences, Tehran, Iran; 2 School of Medicine, Tehran University of Medical Sciences, Tehran, Iran; 3 General Surgery Department, Tehran University of Medical Sciences, Tehran, Iran; 4 School of Medicine, Isfahan University of Medical Sciences, Isfahan, Iran; 5 Students Research Committee, School of Medicine, Shahid Beheshti University of Medical Sciences, Tehran, Iran; 6 Faculty of Medicine, Bogomolets National Medical University (NMU), Kyiv, Ukraine; 7 Guilan University of Medical Sciences, Rasht, Iran; 8 School of Medicine, Shahid Beheshti University of Medical Sciences, Arabi Ave, Daneshjoo Blvd, Velenjak, Tehran, 19839-63113 Iran

**Keywords:** Alcohol use disorder, GLP-1 receptor agonists, Diabetes, Alcohol, Meta-analysis

## Abstract

**Background:**

Alcohol use disorder (AUD) affects nearly half a billion people globally and is associated with significant physical and psychiatric comorbidities. Glucagon-like peptide-1 receptor agonists (GLP-1RAs), approved for diabetes and obesity, have shown promise in modulating reward-related brain pathways, suggesting potential benefits in the management of AUD.

**Methods:**

This systematic review and meta-analysis, registered in PROSPERO and conducted per PRISMA guidelines, assessed the effects of GLP-1RA use on AUD and alcohol-related outcomes in adults with obesity or type 2 diabetes mellitus. Five databases (PubMed, Embase, Web of Science, Scopus, and Cochrane Library) were searched up to September 30, 2025. Random-effects models were applied, and sensitivity analyses examined result stability. No subgroup or meta-regression analyses were performed owing to the small number of eligible studies.

**Results:**

Five observational cohort studies (three on AUD diagnosis, two on alcohol-related hospitalization) were included, with sample sizes ranging from 4,321 to > 53,000 participants. GLP-1RA use was associated with a 28% lower risk of AUD diagnosis (HR = 0.72, 95% CI 0.59–0.89; I² = 65%). For alcohol-related hospitalization, a non-significant reduction was observed (HR = 0.76, 95% CI 0.57–1.01; I² = 77%). Leave-one-out sensitivity analyses confirmed the direction and magnitude of the AUD finding but highlighted the limited evidence base for hospitalization.

**Conclusion:**

GLP-1RA use was associated with a reduced risk of AUD diagnosis, with a possible but non-significant reduction in alcohol-related hospitalization. Effects may be mediated through modulation of mesolimbic reward pathways and the gut–brain axis. Further large-scale trials are warranted to confirm these findings.

**Graphical abstract:**

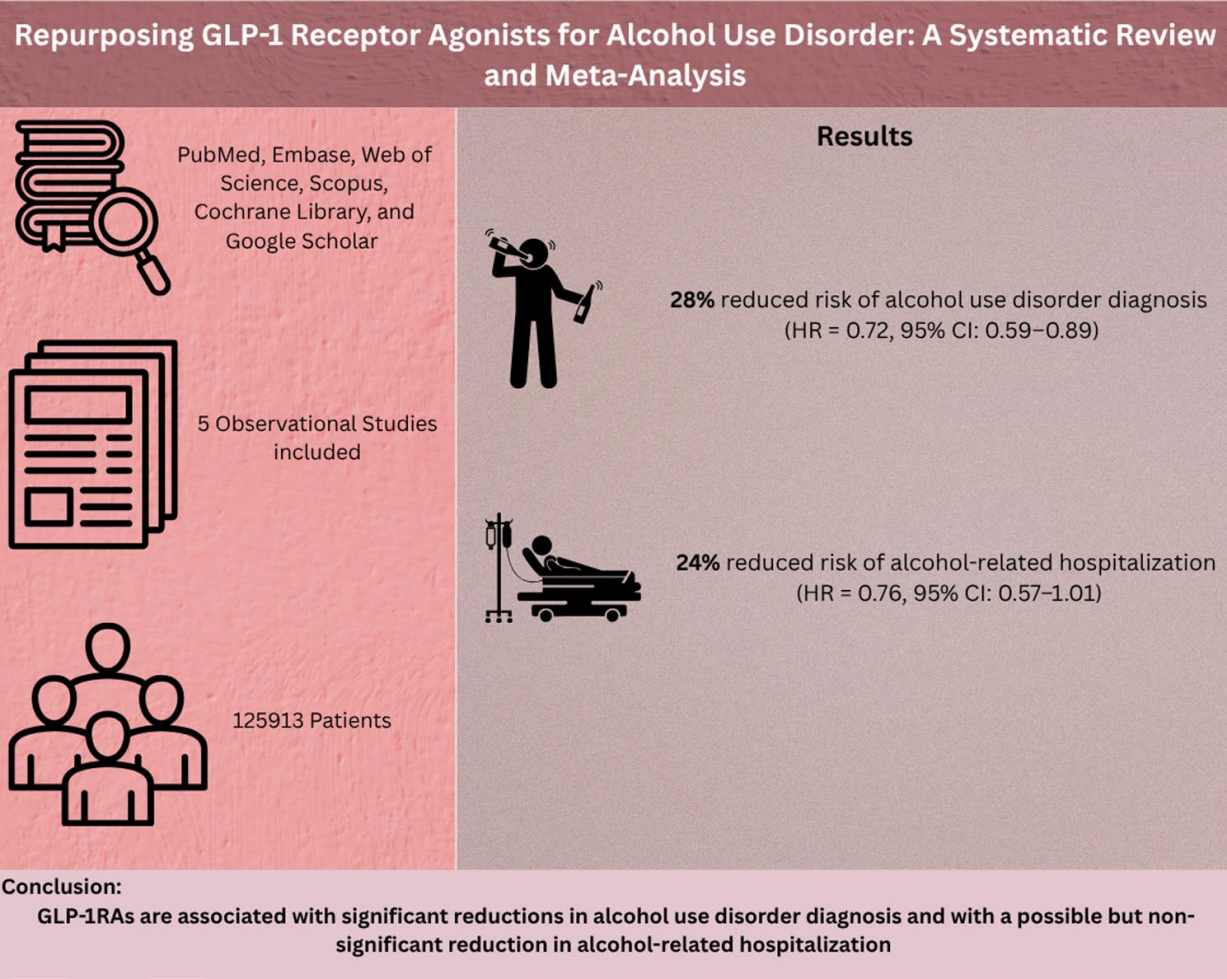

## Introduction

Alcohol misuse and alcohol use disorder (AUD) pose a significant global health challenge, characterized by impaired control over alcohol use, compulsive consumption, and negative affect during withdrawal, which contribute to relapse [1]. AUD is a serious cause of mortality and morbidity, with more than 400 million people around the world affected by their inability to manage their alcohol consumption [2, 3]. There are also many disabilities and medical conditions causally associated with alcohol, with more prevalent conditions (such as cardiovascular diseases, liver disorders, malignancies, and other related conditions) contributing to a significant amount of alcohol-associated morbidity [4].

The treatment of AUD includes psychosocial and pharmacological interventions [5]. Psychosocial treatments are the cornerstone of AUD management, though pharmacological options are also beneficial [6]. To date, only a limited number of medications (disulfiram, acamprosate, and naltrexone) have been approved for the treatment of AUD by the Food and Drug Administration (FDA) and the European Medicines Agency (EMA). A fourth medication, nalmefene, is currently approved by the EMA but not by the FDA [7, 8]. Although the aforementioned medications demonstrate some efficacy, their number is limited, and not all patients respond to them. Therefore, expanding the range of available pharmacotherapies is essential to better support individuals with AUD [9]. There is a critical need to identify new molecular targets to expand the range of treatment options for AUD. Although current therapies benefit some individuals, relapse rates remain high, with most studies reporting rates exceeding 70% within one year, underscoring the necessity for more effective interventions [10, 11].

Endogenous glucagon-like peptide-1 (GLP-1) is a 30-amino acid peptide hormone synthesized by intestinal L cells in response to nutrient intake, as well as in the nucleus tractus solitarius of the medulla oblongata [12, 13]. GLP-1 stimulates insulin secretion, inhibits glucagon release, and, perhaps most notably, suppresses appetite and reduces food intake [14]. Although GLP-1 receptor agonists (GLP-1RAs) were initially developed for the management of diabetes and weight loss, their therapeutic applications have since broadened considerably to encompass a range of cardiovascular, renal, and metabolic disorders [15–17]. Preliminary investigations have also suggested the potential therapeutic benefits of this drug class in the treatment of AUD [18]. These agents appear to modulate brain regions implicated in addiction, thereby reducing alcohol cravings and consumption, particularly among individuals with obesity [19, 20]. Given that alcohol and drug abuse activate the same reward pathways involved in food intake, it is plausible that appetite-regulating peptides, such as GLP-1, exert their effects on brain regions associated with reward and addiction [20, 21]. Their efficacy in treating both AUD and metabolic disorders, including obesity and type 2 diabetes mellitus (T2DM), offers a compelling additional clinical rationale for their use [22, 23].

GLP-1 and its receptor agonists appear to modulate brain regions implicated in addiction, particularly the mesolimbic reward system, which also plays a central role in the regulation of food and substance use. These agents are believed to suppress presynaptic dopamine release and dampen postsynaptic reward signaling at mesolimbic synapses, thereby diminishing the reinforcing effects of alcohol consumption. Given the substantial overlap between the neural circuits governing appetite and those involved in addictive behaviors, it is plausible that appetite-regulating peptides such as GLP-1 contribute to reduced alcohol cravings and intake, an effect that has been especially evident in individuals with obesity.

Despite encouraging preclinical findings and limited clinical evidence, the therapeutic impact of GLP-1RAs on AUD remains unclear. Given their ability to modulate reward pathways shared by both food and substance use (particularly the mesolimbic dopamine system), GLP-1RAs may offer a novel approach to reducing alcohol cravings and consumption. However, the clinical efficacy of GLP-1RAs in AUD has yet to be clearly established. To the best of our knowledge, this study represents the first meta-analysis specifically designed to systematically evaluate the clinical effects of GLP-1RAs on AUD and alcohol-related hospitalization.

## Methods

### Protocol and registration

The present systematic review and meta-analysis was conducted in accordance with the Preferred Reporting Items for Systematic Reviews and Meta-Analyses (PRISMA) guidelines to ensure methodological rigor and transparency. The review aimed to evaluate the impact of GLP-1RAs specifically on alcohol use disorder (AUD; incidence and/or recurrence) and alcohol-related hospitalization in adults with obesity or T2DM. The protocol was registered with the International Prospective Register of Systematic Reviews (PROSPERO) under the registration number CRD420251039831. As this study was based exclusively on previously published data, ethical approval was not required. Nonetheless, all procedures adhered to best practices for systematic reviews involving observational data.

### Search strategy and study selection

We conducted a comprehensive systematic search across five electronic databases: PubMed, Embase, Web of Science, Scopus, and the Cochrane Library, as well as the Google Scholar search engine, from inception to September 30, 2025, to identify studies investigating the association between GLP-1 receptor agonists and AUD outcomes. The search strategy incorporated terms related to GLP-1 receptor agonists (e.g., “GLP-1,” “Semaglutide,” “Liraglutide,” “Exenatide”) in combination with alcohol use concepts (e.g., “alcohol,” “alcohol use disorder,” “binge drinking,” “ethanol”). No restrictions were applied regarding language, publication date, or study design during the initial search. Additional relevant studies were identified through manual screening of the reference lists of included articles and pertinent review papers.

### Inclusion and exclusion criteria

The following PICOS criteria guided study inclusion:

- Population: Adults with obesity or T2DM, evaluated for incident or recurrent AUD.
- Intervention: Treatment with any GLP-1RA (e.g., Semaglutide, liraglutide, dulaglutide).
- Comparison: Studies comparing GLP-1RA therapy to placebo, no GLP-1RA, or other active comparators (e.g., DPP-4 inhibitors).
- Outcomes: AUD diagnosis and/or alcohol-related hospitalization, preferentially reported as hazard ratios (HRs) with 95% confidence intervals (CIs).
- Study Design: Randomized controlled trials and observational cohort studies were included. Conference abstracts and preprints were not included due to insufficient methodological detail. Case reports, reviews, and editorials were excluded.

### Data extraction

Two reviewers independently screened the titles and abstracts of the retrieved records to identify potentially eligible studies (S.J. and S.K.). Full texts of the selected articles were subsequently assessed for inclusion, with exclusion reasons documented. Data were extracted using a standardized form, and the process was conducted independently by both reviewers to ensure accuracy and minimize bias. Any disagreements were resolved through discussion, with a third reviewer consulted when necessary. Key study characteristics were extracted, including first author, publication year, country, study design, GLP-1RA type, comparator group, outcome(s) of interest, follow-up duration, sample size, mean age, standard deviation (if available), and percentage of male participants. For quantitative synthesis, effect estimates were collected when available, including HRs, 95% CI, and associated p-values. Additional data were gathered on covariate adjustment strategies (e.g., age, sex, and metabolic disorders) and relevant medical histories (e.g., obesity, type 2 diabetes mellitus). When outcomes were reported at multiple time points, the most recent follow-up data were extracted to maintain consistency across studies.

### Quality assessment

The methodological quality of the included studies was evaluated using the Newcastle-Ottawa Scale (NOS) [24], which assesses three domains: selection of study participants, comparability between groups, and the ascertainment of exposure or outcomes, with a maximum achievable score of 9 points. Two reviewers independently conducted the quality assessments, and any discrepancies were resolved through discussion or consultation with a third reviewer (A.N. and G.K). Scores ≥ 7 were considered low risk of bias, 4–6 moderate, and ≤ 3 high risk. Because all included studies were observational cohorts, results were interpreted cautiously; residual confounding and selection bias remain possible. Accordingly, we describe overall quality as “low-to-moderate risk of bias.”

### Outcome definitions and pooling

All outcomes were based on ICD-10 F10 codes. De Giorgi et al. [25] and Wang et al. [26], and Adeniran et al. [27] reported diagnostic endpoints (first or recurrent AUD diagnoses), which we pooled as an AUD diagnosis. Lähteenvuo et. al [28] and Al-Moussally et al. [29] reported inpatient admissions with ICD-10 F10 codes, which we pooled as alcohol-related hospitalization. The study by Adeniran et al. [27] was conducted specifically among patients who had undergone Roux-en-Y gastric bypass (RYGB); it therefore represents a post-surgical population contributing only to the AUD diagnosis analysis.

### Statistical analysis

All statistical analyses were conducted using R (version 4.4.2), with the metafor package used for meta-analytic modeling. For each included study, we extracted HRs with corresponding 95% CIs for outcomes of interest: AUD diagnosis and alcohol-related hospitalization. Reported HRs were log-transformed for analysis, and standard errors were derived from the reported CIs.

When studies reported multiple GLP-1RAs, we prespecified the inclusion of Semaglutide to ensure comparability across studies and minimize heterogeneity. In contrast, Al-Moussally et al. [29] did not report separate results by individual GLP-1RA, and therefore, the overall GLP-1RA group was included. Each independent comparison was treated as a separate data point.

Random-effects models (restricted maximum likelihood, REML) were fitted to estimate pooled log-HRs, which were then back-transformed for interpretability. Between-study heterogeneity was assessed using the Q statistic, τ² (tau-squared), and I² statistic. Sensitivity analyses were conducted using leave-one-out diagnostics to examine the influence of individual studies on the overall pooled effect.

Given the small number of studies available for each outcome (k = 3 for AUD; k = 2 for hospitalization), no subgroup analyses or meta-regression were performed, as these approaches are not reliable with very few studies.

## Results

### Study selection

**Fig. 1:**
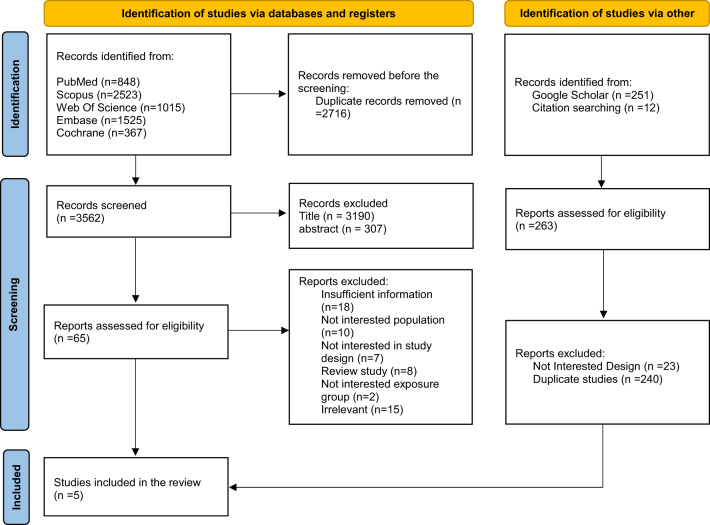
PRISMA Flow Diagram of Study Selection Process. PRISMA flow diagram illustrating the systematic process of study identification, screening, and inclusion. A total of 6,541 records were identified across databases, including PubMed (*n* = 848), Scopus (*n* = 2,523), Web of Science (*n* = 1,015), Embase (*n* = 1,525), and Cochrane Library (*n* = 367), along with 263 additional records identified through Google Scholar (*n* = 251) and citation searching (*n* = 12). After removing 2,716 duplicate records, 3,825 unique studies were screened by title and abstract, resulting in the exclusion of 3,760 articles that did not meet eligibility criteria. A total of 65 reports were assessed for eligibility, of which 60 were excluded due to insufficient information (*n* = 18), non-relevant population (*n* = 10), unsuitable study design (*n* = 7), review articles (*n* = 8), unrelated exposure group (*n* = 2), and irrelevance to the topic (*n* = 15). Additionally, 263 reports identified through other sources were reviewed for eligibility and excluded due to unsuitable design (*n* = 23) or duplication (*n* = 240). Ultimately, five studies met all inclusion criteria and were incorporated into the final systematic review and quantitative synthesis.

The study selection process is illustrated in Fig. 1. A comprehensive literature search across multiple databases identified a total of 6,541 studies, retrieved from PubMed (*n* = 848), Web of Science (*n* = 1,015), Scopus (*n* = 2,523), Embase (*n* = 1,525), Cochrane (*n* = 367), as well as Google Scholar and manual reference searches (*n* = 263). After removing 2,716 duplicate records, 3,825 studies underwent title and abstract screening. Of these, 3,760 articles were excluded based on predefined eligibility criteria, leaving 65 full-text studies for further assessment. Subsequently, 60 studies were excluded due to the absence of alcohol-related outcomes, lack of GLP-1RA exposure, or unsuitable study design. Five studies were included in the final review and quantitative synthesis.

### Study characteristics

Characteristics of the included studies are presented in Table 1. All five were observational cohort studies. Sample sizes ranged from 4,321 to more than 53,000 participants per arm. Mean age ranged from 47.8 to 61 years when reported; the proportion of male participants ranged from 17.7% to 94.7%. Follow-up ranged from 12 months to 9 years. Exposures included semaglutide [25, 26, 28] and the overall GLP-1RA class [27, 29]. Outcomes were ascertained from routinely collected health records (e.g., registers, EHR, claims). Three studies reported adjusted HRs for AUD diagnosis, while two studies reported adjusted HRs for alcohol-related hospitalization.

**Table 1:** Characteristics of included studies in the study

Author	Year	Country	Study design	Sample size	Mean age (SD)	Male (%)	Follow up duration	GLP-1RA agent(s)	Comparator	Outcome	Medical History (T2DM/Obesity)
Adeniran et al. [27]	2025	Multinational	Retrospective cohort	6876	78.5	17.7	4.0 to13.3 years	Liraglutide, Semaglutid, Dulaglutide, Tirzepatide	Non- GLP-1RA	AUD	Post-RYGB (obesity)
Al-Moussally et al. [29]	2025	USA	Retrospective cohort	14,604	61 (10)	94.7	15 years	Liraglutide, Semaglutid, Dulaglutide, Exenatide, Albiglutide, Lixisenatide	DPP4i	Hospitalization	T2DM
De Giorgi et al. [25]	2024	USA	Retrospective cohort	46,772	56.7 (12.2)	43.5	Up to 2 years	Semaglutide	DPP4i	AUD	T2DM
Lähteenvuo et al. [28]	2025	Multinational	Retrospective cohort	4321	NR	NR	At least 1 year	Semaglutide	Within -subject (non - use)^a^	Hospitalization	Obesity, T2DM
Wang et al. [26]	2024	USA	Retrospective cohort	53,340	51.2 (1‍3.2)	28.8	12 months to 3 years	Semaglutide	Non- GLP-1RA	AUD	Only T2DM subgroup

GLP-1RA: glucagon-like peptide-1 receptor agonist, SD: standard deviation, AUD: alcohol use disorder, D: type 2 diabetes mellitus, NR: not reported, DPP-4i: dipeptidyl peptidase-4 inhibitor, RYGB: Roux-en-Y gastric bypass

a: Lähteenvuo et al. used a within-individual design comparing GLP-1RA exposure vs. non-exposure periods in the same patients, without a separate control group

### Alcohol use disorder (AUD)

**Fig. 2:**
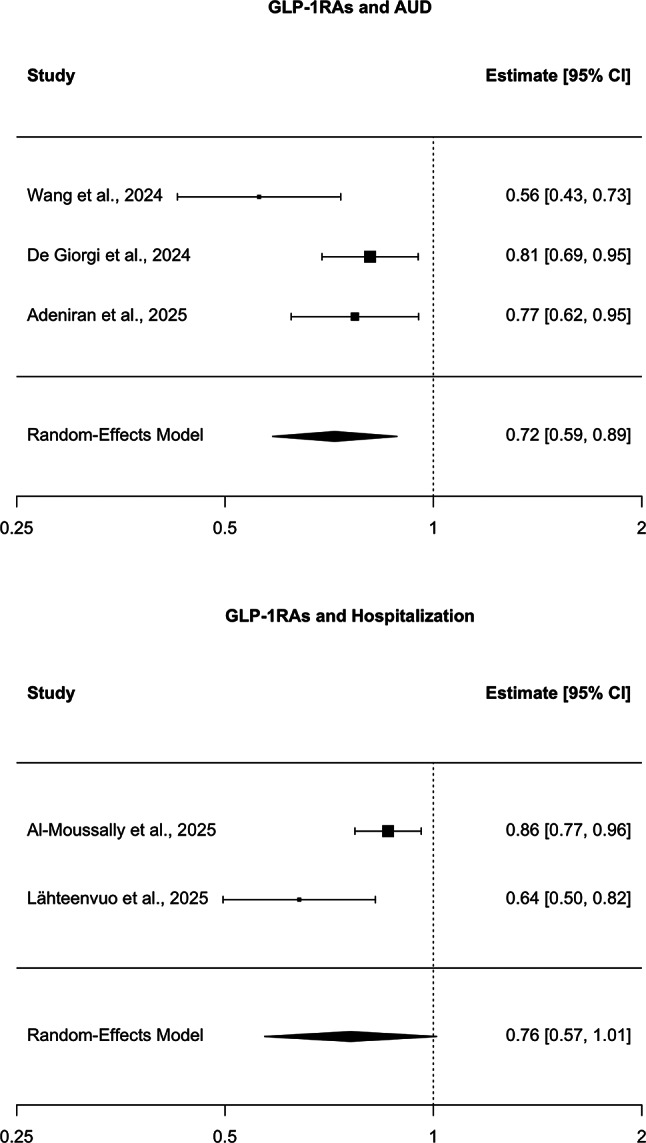
Forest plots of GLP-1 receptor agonists and alcohol-related outcomes. (**A**) Alcohol use disorder (AUD): Random-effects meta-analysis of three retrospective cohort studies. Pooled hazard ratio (HR) = 0.72 (95% CI: 0.59–0.89; *p* = 0.0019), indicating a statistically significant reduction in risk with GLP-1RA use. Heterogeneity was moderate (I² = 65%). (**B**) Alcohol-related hospitalization: Random-effects meta-analysis of two retrospective cohort studies. Pooled HR = 0.76 (95% CI: 0.57–1.01; *p* = 0.059), suggesting a possible protective effect of GLP-1RAs, though not statistically significant. Heterogeneity was high (I² = 77%). HRs < 1.0 favor GLP-1RA exposure. CI = confidence interval; REML = restricted maximum likelihood estimator.

A random-effects meta-analysis of three studies [25–27] demonstrated that GLP-1RA use was associated with a significantly lower risk of AUD diagnosis compared with non–GLP-1RA comparators (HR = 0.72, 95% CI: 0.59–0.89, *p* = 0.0019). Between-study heterogeneity was moderate (I² = 65%, Q = 5.39, *p* = 0.068; τ² = 0.0217). Leave-one-out sensitivity analysis showed that while each study had a measurable influence, the protective association remained consistent in direction and magnitude (Fig. 2A).

### Alcohol-related hospitalization

For alcohol-related hospitalization [28, 29], the pooled HR was 0.76 (95% CI: 0.57–1.01, *p* = 0.059), suggesting a possible protective effect of GLP-1RAs, though not statistically significant. Heterogeneity was high (I² = 77%, Q = 4.39, *p* = 0.036; τ² = 0.034). Leave-one-out analyses indicated that results were largely driven by single-study influence, reflecting the limited evidence base (Fig. 2B).

### Qualitative data assessment

A summary of study quality is shown in Table 2. Using the NOS, the three AUD cohorts were rated low-to-moderate risk of bias, while one study (Qeadan et al. [30]) was rated moderate risk due to potential follow-up limitations.

**Table 2:** Study quality was assessed using the Newcastle-Ottawa scale (NOS)

Study	Selection (4)	Comparability (2)	Outcome (3)	Total score (9)	Quality
Adeniran et al. [27]	+++	++	++	7	Moderate risk
Al-Moussally et al. [29]	+++	++	++	7	Moderate risk
De Giorgi et al. [25]	+++	++	++	7	Moderate risk
Lähteenvuo et al. [28]	++++	++	++	8	Low risk
Wang et al. [26]	++++	++	++	8	Low risk

Scores ≥ 7 indicate low risk of bias. + indicates a point awarded per domain criterion

## Discussion

This systematic review and meta-analysis evaluated the association between GLP-1RA use and the risk of AUD in adults with obesity or T2DM. We restricted the scope to ICD-10–defined outcomes and analyzed AUD diagnoses and alcohol-related hospitalization separately. Across three observational cohort studies, GLP-1RA exposure was associated with a 28% lower risk of AUD diagnosis. Between-study heterogeneity was substantial, reflecting variability in design and comparator groups. Because only three cohorts were available, no formal meta-regression was performed. Observed heterogeneity likely reflects differences in comparator drugs (DPP-4 inhibitors vs. no GLP-1RA), baseline risk, and follow-up duration rather than a single identifiable source. It may also arise from the inclusion of the Adeniran et al. [27] cohort, which involved patients who had undergone Roux-en-Y gastric bypass (RYGB), a bariatric procedure known to carry a higher risk of AUD and alcohol-related complications [31, 32]. These findings align with emerging research on GLP-1RAs as novel pharmacotherapies for AUD, a condition with limited effective treatments [33]. For alcohol-related hospitalization, pooling two cohorts suggested a possible protective effect, but this did not reach statistical significance. The trend warrants further investigation, as even modest reductions in hospitalization could have important clinical and economic implications if confirmed in larger datasets. The observed reduction in AUD risk is particularly noteworthy given the global burden of AUD, which affects an estimated 280 million people and is associated with high morbidity and mortality [34].

These findings are supported by previous evidence from randomized controlled trials, although results remain mixed. One trial found that exenatide had no overall benefit on alcohol consumption in individuals with AUD, except in participants with obesity [23]. Another study, using dulaglutide, reported a medium-to-large effect size for reducing alcohol use in people with obesity, although no benefit was seen in heavy drinkers [35]. These different outcomes may reflect variations in GLP-1RA agent, treatment duration, underlying conditions, and baseline alcohol consumption.

Because the hospitalization outcome included only two studies and showed high heterogeneity, these findings should be interpreted cautiously.

In addition to our included studies, there were more original studies excluded from our meta-analysis due to methodological differences and a lack of appropriate data, which provide additional support for GLP-1RAs’ role in reducing alcohol consumption. In a 26-week Randomized Controlled Trial (RCT), Klausen et al. [23] evaluated exenatide in individuals with AUD. While the overall reduction in heavy drinking days did not reach significance, exploratory analyses showed notable reductions in alcohol intake among participants with obesity. In contrast, exenatide appeared to worsen drinking outcomes in lean individuals, suggesting that body weight may influence treatment response. Neuroimaging data from the same study indicated reduced alcohol cue reactivity in reward-related brain regions and decreased dopamine transporter availability, offering a plausible neurobiological mechanism for these effects [23].

In the study by Jensen et al. [36], alcohol consumption was measured using the biomarker phosphatidylethanol (PEth). A significant reduction in PEth levels was observed at week 26 in the exenatide group compared to placebo, though no differences emerged earlier. This delayed effect may relate to exenatide’s pharmacokinetics or the time needed to influence compulsive drinking behaviors [37]. Moreover, O’Farrell et al. [38] suggested that obese patients treated with liraglutide or Semaglutide showed a marked reduction in self-reported alcohol intake after 3–6 months, particularly among high consumers. Weight loss was also observed, with a modest correlation between alcohol reduction and weight change, suggesting GLP-1RAs may simultaneously target both alcohol use and obesity.

Other original studies, such as those included in a recent systematic review, reported benefits with Semaglutide and dulaglutide [39]. For example, Probst et al. conducted a secondary analysis of an RCT using dulaglutide in smokers with AUD, finding trends toward reduced alcohol use, but data limitations precluded inclusion in our meta-analysis [35]. Case series, such as Richards et al., also reported decreased AUD symptoms based on alcohol use disorder identification test score in patients receiving Semaglutide for weight loss, further corroborating the potential of GLP-1RAs across formulations [40]. Together, these findings illustrate the consistency of alcohol-reducing effects across real-world, biomarker-based, and neuroimaging-supported studies, despite heterogeneity in design and outcome measures.

The efficacy of GLP-1RAs in AUD likely stems from their modulation of the brain’s reward circuitry, as evidenced by preclinical and clinical data [41, 42]. GLP-1 receptors are expressed in reward-related regions, such as the nucleus accumbens (NAc) and ventral tegmental area (VTA), where they influence dopaminergic signaling [43]. Preclinical studies in rodent models demonstrate that GLP-1RAs, including exenatide, liraglutide, and semaglutide, attenuate alcohol-induced dopamine release in the NAc, reducing the reinforcing properties of alcohol [44]. For instance, a study found that semaglutide modulates central GABA neurotransmission, which is implicated in alcohol withdrawal and craving [45]. In an experimental study conducted on African vervet monkeys, administration of liraglutide and exenatide led to a significant reduction in alcohol intake without any signs of emesis [46]. Similarly, another preclinical investigation examined the effects of semaglutide on voluntary alcohol consumption in non-human primates and demonstrated a marked decrease in alcohol intake, with no evidence of emetic responses [47]. Although these animal models (such as those employing chronic intermittent ethanol exposure paradigms) provide compelling mechanistic insights, extrapolation to human populations should be approached with caution, given interspecies physiological and behavioral differences. Additionally, GLP-1RAs promote satiety and delay gastric emptying, potentially decreasing the drive for alcohol as part of broader ingestive behavior changes [41]. Also, in alcohol-dependent rodents, administration of GLP-1R agonists mitigated signs of alcohol withdrawal and associated anxiety-like behavior [48]. This dual effect on both positive and negative reinforcement mechanisms suggests that GLP-1RAs may reduce alcohol use through multiple neurobiological pathways. Genetic studies further support a biological link through the gut-brain axis, identifying associations between GLP-1 receptor gene variants and AUD [49].

The primary strength of this meta-analysis lies in its novelty and methodological rigor. While previous studies have provided systematic or narrative reviews examining the relationship between GLP-1RAs and alcohol use, this work represents the first quantitative meta-analysis to synthesize effect estimates across observational cohorts. The analysis was conducted in strict accordance with PRISMA guidelines and employed a random-effects model to account for interstudy heterogeneity. By utilizing HRs as the effect measure, we ensured greater comparability among studies and enhanced the robustness and stability of the pooled estimate for the association between GLP-1RAs and AUD. However, we acknowledge that our research may have some limitations. First, the small number of included studies may affect the overall quality of our pooled analysis; however, this reflects the early stage of research in this field. Notably, most studies were published very recently (2024–2025), highlighting the novelty of this topic and the emerging nature of GLP-1 research in this context. The absence of RCTs among the included studies is a notable limitation, as observational and retrospective designs may be more susceptible to confounding, selection bias, and variability in data quality. This limitation highlights the inherent difficulties of performing a meta-analysis that relies exclusively on retrospective cohorts. High heterogeneity suggests variability in study populations or methodologies, which may partly stem from differences in study design, follow-up duration, and outcome assessment. Although we sought to further elucidate the sources of heterogeneity, subgroup analyses based on sex, BMI, or other covariates could not be performed because too few studies and insufficient data were available. Additionally, different studies use various measurement criteria for reporting their outcomes, which hinders our ability to conduct optimal analysis. Moreover, each study used different time frames for follow-up, which adds to the heterogeneity of our results and makes it impossible to assess the long-term outcomes. Future RCTs should test various GLP-1RAs across broader AUD populations, standardize outcomes, and explore body weight’s moderating role. Long-term studies on relapse prevention and combination therapies (e.g., with naltrexone) are also critical.

## Conclusion

This meta-analysis suggests that GLP-1RAs, beyond their metabolic benefits, are associated with reduced risks of AUD. These effects are likely mediated through modulation of the brain’s reward system and gut–brain axis. The benefits were more pronounced when compared to no treatment, and findings remained robust across sensitivity analyses. While heterogeneity and limited study numbers warrant caution, these results highlight GLP-1RAs as promising candidates for AUD management. Further large-scale, long-term trials are needed to confirm these effects.

## Data Availability

All data relevant to the findings are included in the manuscript. Additional datasets analyzed during the current study are available from the corresponding author upon reasonable request.

## Abbreviations

AUD: Alcohol Use Disorder
CI: Confidence Interval
DPP-4: Dipeptidyl Peptidase-4
DPP-4i: Dipeptidyl Peptidase-4 Inhibitor
EMA: European Medicines Agency
FDA: Food and Drug Administration
GABA: Gamma-Aminobutyric Acid
GLP-1: Glucagon-Like Peptide-1
GLP-1RA: Glucagon-Like Peptide-1 Receptor Agonist
HR: Hazard Ratio
I²: I-squared Statistic
ICD-10: International Classification of Diseases, 10th Revision
NAc: Nucleus Accumbens
NOS: Newcastle–Ottawa Scale
NR: Not Reported
PEth: Phosphatidylethanol
PICOS: Population, Intervention, Comparison, Outcomes, Study Design
PRISMA: Preferred Reporting Items for Systematic Reviews and Meta-Analyses
PROSPERO: International Prospective Register of Systematic Reviews
Q: Cochran’s Q Statistic
RCT: Randomized Controlled Trial
REML: Restricted Maximum Likelihood
RYGB: Roux-en-Y Gastric Bypass
SD: Standard Deviation
SE: Standard Error
T2DM: Type 2 Diabetes Mellitus
τ²: Tau-squared
VTA: Ventral Tegmental Area

## References

[1] Carvalho AF, Heilig M, Perez A, Probst C, Rehm J. Alcohol use disorders. Lancet. 2019;394(10200):781–92. 31478502 10.1016/S0140-6736(19)31775-1

[2] Schuckit MA. Alcohol-use disorders. Lancet. 2009;373(9662):492–501. 19168210 10.1016/S0140-6736(09)60009-X

[3] The Lancet G, amp. Hepatology. Global progress towards alcohol harm reduction insufficient. Lancet Gastroenterol Hepatol. 2024;9(9):773. 10.1016/S2468-1253(24)00239-5 39127066

[4] Smyth A, Teo KK, Rangarajan S, O’Donnell M, Zhang X, Rana P, et al. Alcohol consumption and cardiovascular disease, cancer, injury, admission to hospital, and mortality: a prospective cohort study. Lancet. 2015;386(10007):1945–54. 26386538 10.1016/S0140-6736(15)00235-4

[5] Peacock A, Eastwood B, Jones A, Millar T, Horgan P, Knight J, et al. Effectiveness of community psychosocial and pharmacological treatments for alcohol use disorder: a national observational cohort study in England. Drug Alcohol Depend. 2018;186:60–7. 29550623 10.1016/j.drugalcdep.2018.01.019

[6] Coriale G, Fiorentino D, De Rosa F, Solombrino S, Scalese B, Ciccarelli R, et al. Treatment of alcohol use disorder from a psychological point of view. Rivista Di Psichiatria. 2018;53(3):141–8. 29912216 10.1708/2925.29416

[7] McPheeters M, O’Connor EA, Riley S, Kennedy SM, Voisin C, Kuznacic K, et al. Pharmacotherapy for alcohol use disorder: a systematic review and meta-analysis. JAMA. 2023;330(17):1653–65. 37934220 10.1001/jama.2023.19761 PMC10630900

[8] Mann K, Torup L, Sørensen P, Gual A, Swift R, Walker B, et al. Nalmefene for the management of alcohol dependence: review on its pharmacology, mechanism of action and meta-analysis on its clinical efficacy. Eur Neuropsychopharmacol. 2016;26(12):1941–9. 27842940 10.1016/j.euroneuro.2016.10.008

[9] Kranzler HR, Soyka M. Diagnosis and pharmacotherapy of alcohol use disorder: a review. JAMA. 2018;320(8):815–24. 30167705 10.1001/jama.2018.11406 PMC7391072

[10] Heilig M, Egli M. Pharmacological treatment of alcohol dependence: target symptoms and target mechanisms. Pharmacol Ther. 2006;111(3):855–76. 16545872 10.1016/j.pharmthera.2006.02.001

[11] Ray LA, Bujarski S, Grodin E, Hartwell E, Green R, Venegas A, et al. State-of-the-art behavioral and pharmacological treatments for alcohol use disorder. Am J Drug Alcohol Abuse. 2019;45(2):124–40. 30373394 10.1080/00952990.2018.1528265 PMC6430676

[12] Ahrén B. Glucagon-like peptide‐1 (GLP‐1): A gut hormone of potential interest in the treatment of diabetes. BioEssays. 1998;20(8):642–51. 9780839 10.1002/(SICI)1521-1878(199808)20:8<642::AID-BIES7>3.0.CO;2-K

[13] Holt MK, Richards JE, Cook DR, Brierley DI, Williams DL, Reimann F, et al. Preproglucagon neurons in the nucleus of the solitary tract are the main source of brain GLP-1, mediate stress-induced hypophagia, and limit unusually large intakes of food. Diabetes. 2019;68(1):21–33. 30279161 10.2337/db18-0729 PMC6314470

[14] Shah M, Vella A. Effects of GLP-1 on appetite and weight. Rev Endocr Metab Disord. 2014;15:181–7. 24811133 10.1007/s11154-014-9289-5 PMC4119845

[15] Karimi MA, Gholami Chahkand MS, Dadkhah PA, Sheikhzadeh F, Yaghoubi S, Esmaeilpour Moallem F et al. Comparative effectiveness of semaglutide versus liraglutide, dulaglutide or tirzepatide: a systematic review and meta-analysis. Front Pharmacol. 2025;Volume 16–2025. 10.3389/fphar.2025.1438318 PMC12120964 40444045

[16] Ndumele CE, Rangaswami J, Chow SL, Neeland IJ, Tuttle KR, Khan SS, et al. Cardiovascular-Kidney-Metabolic health: a presidential advisory from the American Heart Association. Circulation. 2023;148(20):1606–35. 37807924 10.1161/CIR.0000000000001184

[17] Theodorakis N, Nikolaou M. Integrated management of Cardiovascular–Renal–Hepatic–Metabolic syndrome: expanding roles of SGLT2is, GLP-1 RAs, and GIP/GLP-1 RAs. Biomedicines. 2025;13(1):135. 39857719 10.3390/biomedicines13010135 PMC11760485

[18] Litten RZ, Wilford BB, Falk DE, Ryan ML, Fertig JB. Potential medications for the treatment of alcohol use disorder: an evaluation of clinical efficacy and safety. Subst Abuse. 2016;37(2):286–98. 10.1080/08897077.2015.1133472 26928397

[19] Alessi JP. Brain responses to sugar: implications for alcohol use disorder and obesity. Indiana University-Purdue University Indianapolis. 2024.

[20] Jerlhag E. Gut-brain axis and addictive disorders: a review with focus on alcohol and drugs of abuse. Pharmacol Ther. 2019;196:1–14. 30439457 10.1016/j.pharmthera.2018.11.005

[21] Dalton M, Buckland N, Blundell J. Psychobiology of obesity: eating behavior and appetite control. Clin Obes Adults Child. 2022;99–112.

[22] Vancampfort D, Mugisha J, Hallgren M, De Hert M, Probst M, Monsieur D, et al. The prevalence of diabetes mellitus type 2 in people with alcohol use disorders: a systematic review and large scale meta-analysis. Psychiatry Res. 2016;246:394–400. 27788459 10.1016/j.psychres.2016.10.010

[23] Klausen MK, Jensen ME, Møller M, Le Dous N, Jensen A-M, Zeeman VA, et al. Exenatide once weekly for alcohol use disorder investigated in a randomized, placebo-controlled clinical trial. JCI Insight. 2022;7(19):e159863. 36066977 10.1172/jci.insight.159863 PMC9675448

[24] Wells GA, Shea B, O’Connell D, Peterson J, Welch V, Losos M et al. The Newcastle-Ottawa Scale (NOS) for assessing the quality of nonrandomised studies in meta-analyses. 2000.

[25] De Giorgi R, Koychev I, Adler AI, Cowen PJ, Harmer CJ, Harrison PJ, et al. 12-month neurological and psychiatric outcomes of semaglutide use for type 2 diabetes: a propensity-score matched cohort study. eClin Med. 2024. 10.1016/j.eclinm.2024.102726. 10.1016/j.eclinm.2024.102726 PMC11701436 39764175

[26] Wang W, Volkow ND, Berger NA, Davis PB, Kaelber DC, Xu R. Associations of semaglutide with incidence and recurrence of alcohol use disorder in real-world population. Nat Commun. 2024. 10.1038/s41467-024-48780-6. 38806481 10.1038/s41467-024-48780-6 PMC11133479

[27] Adeniran O, Nieto LM, Amadi C, Shepherd K, Kirkpatrick J, Farah K, et al. Alcoholic use disorder outcomes after Roux-en-Y gastric bypass in patients taking GLP-1 ras: a multicenter analysis. Obesity. 2025;33(10):1886–94. 40855971 10.1002/oby.70001

[28] Lähteenvuo M, Tiihonen J, Solismaa A, Tanskanen A, Mittendorfer-Rutz E, Taipale H. Repurposing semaglutide and liraglutide for alcohol use disorder. JAMA Psychiatr. 2025;82(1):94–8. 10.1001/jamapsychiatry.2024.3599 PMC11561716 39535805

[29] Al-Moussally F, Khan S, Katukuri V, Kinaan M, Mansi IA. Association of glucagon-like peptide-1 receptor agonist with progression to liver cirrhosis and alcohol-related admissions in patients with alcohol use disorder and diabetes: a retrospective cohort study. Drugs. 2025;85(6):813–25. 40223043 10.1007/s40265-025-02177-x

[30] Qeadan F, McCunn A, Tingey B. The association between glucose-dependent insulinotropic polypeptide and/or glucagon-like peptide-1 receptor agonist prescriptions and substance-related outcomes in patients with opioid and alcohol use disorders: a real-world data analysis. Addiction. 2025;120(2):236–50. 39415416 10.1111/add.16679 PMC11707322

[31] Azam H, Shahrestani S, Phan K. Alcohol use disorders before and after bariatric surgery: a systematic review and meta-analysis. Ann Transl Med. 2018;6(8):148. 29862237 10.21037/atm.2018.03.16 PMC5952017

[32] Mahmud N, Panchal S, Abu-Gazala S, Serper M, Lewis JD, Kaplan DE. Association between bariatric surgery and alcohol use–related hospitalization and all-cause mortality in a veterans affairs cohort. JAMA Surg. 2023;158(2):162–71. 36515960 10.1001/jamasurg.2022.6410 PMC9856780

[33] Oesterle TS, Ho M-F. Glucagon-like peptide-1 receptor agonists: a new frontier in treating alcohol use disorder. Brain Sci. 2025;15(7):702. 40722294 10.3390/brainsci15070702 PMC12293269

[34] Griswold MG, Fullman N, Hawley C, Arian N, Zimsen SRM, Tymeson HD, et al. Alcohol use and burden for 195 countries and territories, 1990–2016: a systematic analysis for the global burden of disease study 2016. Lancet. 2018;392(10152):1015–35. 30146330 10.1016/S0140-6736(18)31310-2 PMC6148333

[35] Probst L, Monnerat S, Vogt D R, Lengsfeld S, Burkard T, Meienberg A, et al. Effects of dulaglutide on alcohol consumption during smoking cessation. JCI Insight. 2023;8(22):e170419. 37991022 10.1172/jci.insight.170419 PMC10721313

[36] Fineman M, Flanagan S, Taylor K, Aisporna M, Shen LZ, Mace KF, et al. Pharmacokinetics and pharmacodynamics of exenatide extended-release after single and multiple dosing. Clin Pharmacokinet. 2011;50(1):65–74. 21142268 10.2165/11585880-000000000-00000

[37] Jensen ME, Klausen MK, Bergmann ML, Knudsen GM, Vilsbøll T, Stove C, et al. Blood phosphatidylethanol measurements indicate GLP-1 receptor stimulation causes delayed decreases in alcohol consumption. Alcohol Clin Exp Res. 2025. 10.1111/acer.70041. 10.1111/acer.70041 PMC12098802 40123107

[38] O’Farrell M, Almohaileb FI, le Roux CW. Glucagon-like peptide-1 analogues reduce alcohol intake. Diabetes Obes Metabolism. 2025;27(3):1601–4. 10.1111/dom.16152 PMC11802387 39748222

[39] Subhani M, Dhanda A, King JA, Warren FC, Creanor S, Davies MJ, et al. Association between glucagon-like peptide-1 receptor agonists use and change in alcohol consumption: a systematic review. eClin Med. 2024. 10.1016/j.eclinm.2024.102920. 10.1016/j.eclinm.2024.102920 PMC11701477 39764544

[40] Richards JR, Dorand MF, Royal K, Mnajjed L, Paszkowiak M, Simmons WK. Significant decrease in alcohol use disorder symptoms secondary to semaglutide therapy for weight loss: A case series. J Clin Psychiatry. 2023;85(1). 10.4088/JCP.23m15068 38019594

[41] Badulescu S, Tabassum A, Le GH, Wong S, Phan L, Gill H, et al. Glucagon-like peptide 1 agonist and effects on reward behaviour: a systematic review. Physiol Behav. 2024;283:114622. 38945189 10.1016/j.physbeh.2024.114622

[42] Krupa AJ. Curbing the appetites and restoring the capacity for satisfaction: the impact of GLP-1 agonists on the reward circuitry. Neuroscience Applied. 2025;4:105512. 40654594 10.1016/j.nsa.2025.105512 PMC12244148

[43] Bruns Vi N, Tressler EH, Vendruscolo LF, Leggio L, Farokhnia M. IUPHAR review - Glucagon-like peptide-1 (GLP-1) and substance use disorders: an emerging pharmacotherapeutic target. Pharmacol Res. 2024;207:107312. 39032839 10.1016/j.phrs.2024.107312 PMC11467891

[44] Jerlhag E. GLP-1 receptor agonists: promising therapeutic targets for alcohol use disorder. Endocrinology. 2025. 10.1210/endocr/bqaf028. 39980336 10.1210/endocr/bqaf028 PMC11879929

[45] Chuong V, Farokhnia M, Khom S, Pince CL, Elvig SK, Vlkolinsky R, et al. The glucagon-like peptide-1 (GLP-1) analogue semaglutide reduces alcohol drinking and modulates central GABA neurotransmission. JCI Insight. 2023. 10.1172/jci.insight.170671. 37192005 10.1172/jci.insight.170671 PMC10371247

[46] Thomsen M, Holst JJ, Molander A, Linnet K, Ptito M, Fink-Jensen A. Effects of glucagon-like peptide 1 analogs on alcohol intake in alcohol-preferring Vervet monkeys. Psychopharmacology. 2019;236(2):603–11. 30382353 10.1007/s00213-018-5089-z PMC6428196

[47] Fink-Jensen A, Wörtwein G, Klausen MK, Holst JJ, Hartmann B, Thomsen M, et al. Effect of the glucagon-like peptide-1 (GLP-1) receptor agonist semaglutide on alcohol consumption in alcohol-preferring male Vervet monkeys. Psychopharmacology. 2025;242(1):63–70. 38884652 10.1007/s00213-024-06637-2 PMC11742737

[48] Liu W, Wang Z, Wang W, Wang Z, Xing Y, Hölscher C. Liraglutide reduces alcohol consumption, anxiety, memory impairment, and synapse loss in alcohol dependent mice. Neurochem Res. 2024;49(4):1061–75. 38267691 10.1007/s11064-023-04093-6

[49] Jerlhag E. Alcohol-mediated behaviours and the gut-brain axis; with focus on glucagon-like peptide-1. Brain Res. 2020;1727:146562. 31759971 10.1016/j.brainres.2019.146562

